# Quantifying vocal subsystems of adults with minimally verbal autism spectrum disorder

**DOI:** 10.3389/fnhum.2026.1695265

**Published:** 2026-03-23

**Authors:** Thomas F. Quatieri, Tanya Talkar, James R. Williamson, Sophia Yuditskaya, Chakameh Jafari, Meredith Pecukonis, Paige Townsend, Laura Sarnie, Nataliya Kosmyna, Nishat Protyasha, Pattie Maes, Christopher J. McDougle, Lisa Nowinski, Maria Mody

**Affiliations:** 1 MIT Lincoln Laboratory, Lexington, MA, United States; 2 MGH Lurie Center for Autism, Lexington, MA, United States; 3 MGH Martinos Center for Biomedical Imaging, Charlestown, MA, United States; 4 MIT Media Lab, Cambridge, CA, United States

**Keywords:** autism, complexity, dynamics, minimally-verbal, speech

## Abstract

The understanding of speech production and its fine-motor underpinnings in individuals with autism spectrum disorder (ASD) has become an area of growing interest in clinical research. Here, we developed objective acoustic-based measures to characterize and contrast the respiratory, laryngeal, and articulatory vocal production subsystems based on data from 27 adults with minimally-verbal autism spectrum disorder (mv-ASD) relative to 27 age-matched neurotypical (NT) peers. The complexity of representative movement dynamics of each subsystem was determined through signal correlation analysis, associated with degrees of freedom derived from correlation structure. As proxies to underlying speech production, we used base features of signal envelope, pitch and formant trajectories associated with each subsystem, respectively, along with their velocities. In addition, we examined cepstral peak prominence and mel-frequency cepstral coefficients, associated with laryngeal and articulatory systems, widely used in assessing voice and speech in neurological and pathological conditions. With speech data from a protocol consisting of diadochokinetic sequencing (DDK), and three different single-word tasks of varying cognitive load (Imitation, Naming, Reading), we studied complexity of motor dynamics at group and individual levels under each task condition. At group level, mv-ASDs showed lower complexity than NT participants for the respiratory and laryngeal subsystems and, except for the DDK task, higher complexity for the articulatory subsystem. At the individual level, there is a range of complexity consistent with mv-ASD heterogeneity. Using Pearson and Spearman correlation measures, we correlated our subsystem characterization with measures of non-verbal IQ, expressive and receptive vocabulary, visual-motor integration, and fine motor dexterity to understand the clinical relevance of these acoustic features, as well as the effect of outliers. When combining mv-ASD and NT groups, articulatory base features were most consistent in correlating with expressive vocabulary across tasks, compared to respiratory and laryngeal features. The Pearson-based association holds within the mv-ASD group alone for the single-word tasks. Continued understanding of speech and language challenges of individuals with mv-ASD reflected in metrics of each of the three speech production subsystems (respiratory, laryngeal, and articulatory) and their relationship with standardized motor, cognitive, and language assessments, will promote design of functionally-tailored personalized and clinically acceptable interventions.

## Introduction

In the U. S., there are more than 1 million people who are minimally verbal or non-speaking who use little to no spoken language in their day-to-day communication. Our focus here is on individuals with minimally-verbal Autism Spectrum Disorder (mv-ASD) who have less than 30 spoken words used functionally, representing about 30% of the ASD population (Bal et al., 2016; Chenausky et al., 2023; Koegel et al., 2020). To date, there has been little research into characterizing and understanding the vocalizations of this population, though these utterances can be an important tool of communication for these individuals (Mody and McDougle, 2019). Moreover, a characterization of their vocal production has yet to be related to standard clinical assessments. Characterizing the underpinnings of speech difficulties will be essential for designing novel technology for individuals with mv-ASD and guiding personalized intervention.

**Figure 1:**
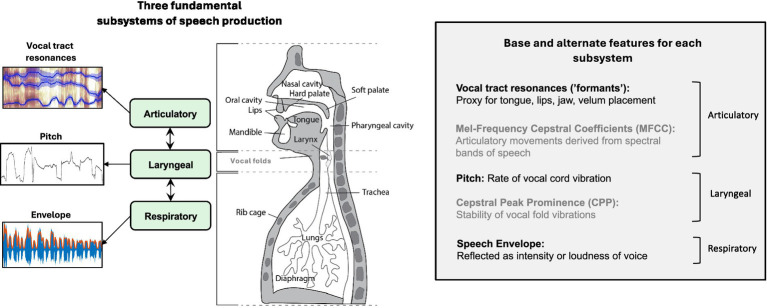
Speech production subsystems. The respiratory subsystem houses the lungs, diaphragm, and trachea, the laryngeal system the vocal folds, and the articulatory system the vocal tract that includes the tongue, lips, jaw, and velum articulators. Movements within each subsystem are coordinated by specific sets of muscles. Base features (right panel in black) comprise: for the respiratory subsystem the *envelope* (proxy for coordinated muscle intensities that are associated with lung pressure and ‘loudness’), for the laryngeal subsystem the *pitc*h or vocal fold vibration frequency (proxy for the activity of muscles surrounding and within the vocal folds), and *formants* or vocal tract resonances (proxy for the muscle activity associated with creation of articulatory constrictions along vocal tract). Features (right panel in gray) providing alternative perspectives include: Mel-frequency cepstral coefficients (MFCCs) and cepstral peak prominence (CPP) features for the articulatory (filtered movements associated with cochlear-like spectral bands) and laryngeal (pitch stability) subsystems, respectively. Anatomical drawing from (Quatieri, 2001; Copyright Pearson Education).

**Figure 2:**
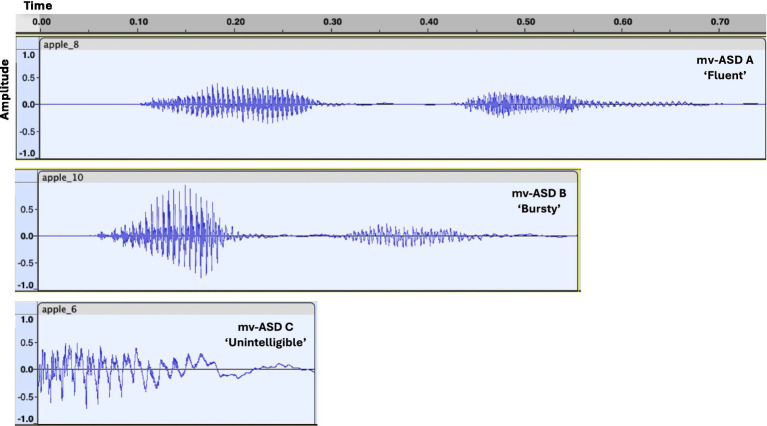
Audio samples from the mv-ASD dataset collected at the MGH Lurie Center for Autism. Examples from 3 different participates illustrate ‘fluent’ (most similar to neurotypicals), ‘bursty’ (with large loudness at word onset), and ‘unintelligible’ (with only a syllable attempt) when asked to imitate the word “apple”.

There are three primary subsystems of speech production: Respiratory, laryngeal, and articulatory (Figure 1), all of which may be affected in ASD. With a lack of motor control, the respiratory system alters its time-varying lung pressure and thus air flow through the trachea. This change can also affect breath timing and word breath grouping. These modifications can take the form of unnatural loudness and burstiness and atypical emotional expression (Hubbard and Trauner, 2007). Decline in laryngeal control can result in irregular vocal fold vibration that creates inappropriate tone, unusual breathy voice quality (Bonneh et al., 2011), but sometimes a ‘flat’ or ‘machine-like’ voice (McCann and Peppé, 2003). Lack of proper coordination of the articulators (tongue, lips, jaw, and velum) can result in slurred or unintelligible sounds (Maffei et al., 2024; Masapollo and Nittrouer, 2023). Figure 2 illustrates the range of acoustic qualities of mv-ASD speech samples from our dataset, manifesting ‘fluent’, ‘bursty’, and ‘unintelligible’ examples when participants are asked to imitate the word “apple.”

This study aimed to develop objective acoustic-based measures to characterize and contrast the respiratory, laryngeal, and articulatory speech production subsystems in adults with mv-ASD relative to age-matched neurotypical (NT) peers. Viewing each subsystem as a dynamical system, the complexity of the movement dynamics of each system (Pinto, 2022) is determined through signal correlation analysis, associated with system degrees of freedom, derived from the correlation structure of speech feature trajectories (Quatieri et al., 2017; Williamson et al., 2019). Motivated by our previous work demonstrating an association between communication deficits and the motor system in ASD (Mody et al., 2017; Talkar et al., 2019; Talkar et al., 2024b), we explore how the notion of complexity of motor dynamics, as used here, differs in an adult mv-ASD sample cohort as compared to an age-matched NT group. We investigate this characterization at both group and individual levels. Our previous work in complexity of speech motor dynamics indicated low complexity in an ASD cohort relative to a matched NT cohort (Talkar et al., 2024b). Nevertheless, due to the difference from our mv-ASD cohort in being a pediatric age range, having higher verbal ability and using a more advanced protocol, as well as due to the known significant heterogeneity within ASD populations, we entered our current study as exploratory, not drawing speculations from our previous work.

A second goal in this exploratory study is to test if differences in our speech production subsystems are related to a set of standardized clinical assessments measuring non-verbal IQ, expressive and receptive vocabulary, visual-motor integration, and fine motor skills, thus providing a clinical grounding to the acoustic-based variables. In another of our previous studies, also with younger individuals on the autism spectrum, 2–17 years (Mody et al., 2017) found significant relationships between standardized test measures of fine motor skills and language ability, consistent with findings in the literature (Bhat et al., 2011; Bhat et al., 2012). Insofar as speech is a sequence of finely tuned phonological gestures (Studdert-Kennedy, 2000), oral motor difficulties in early childhood may set the stage for later speech and language challenges. As such, early motor deficits observed in high-risk infants can have cascading effects on later development of social and communication behaviors, characteristic of ASD. For example, (Iverson and Wozniak, 2007) observed delays in reduplicative babbling and first words onset in infants at high risk for ASD. Of interest to the current study is the relationship between fine motor skills and speech and language specifically in minimally-verbal individuals on the autism spectrum.

## Methods

### Participants

The mv-ASD participants (*N* = 27) and NT participants (*N* = 27) ranged 19–34 years in age, recruited from a variety of sources, including the Lurie Center for Autism’s recruitment database, clinician referrals, local outpatient clinics, primary care practices, autism societies, school and day programs, online advertisements, social media posts, and flyers within the local community. Descriptions of participant demographics are given in Table 1.

**Table 1:** Demographics (age and sex statistics) and mean (sd) of 5 clinical assessments (raw scores) for each group (27 mv-ASD and 27 NT participants) used in analysis. Not all assessments used the full 27 participants in a group (i.e., 1 or 2 less) due to inability to complete a test.

Demographic/assessment	mv-ASD mean (sd)	Controls mean (sd)
Age (years)	25.78 (4.84)	24.22 (3.75)
(range)	19–34	19–33
Sex (female)	11 (41%)	13 (48%)
(male)	16 (59%)	14 (52%)
GPT (seconds)	136.96 (41.67)	65.44 (11.64)
VMI-6	14.00 (5.18)	27.67 (2.12)
Nonverbal (SB-5)	14.81 (6.84)	28.78 (2.49)
PPVT-5	127.42 (27.47)	220.00 (10.01)
EVT-3	69.85 (30.91)	168.08 (10.90)

GPT, grooved pegboard test; VMI-6, visual motor integration; SB-5, Stanford Binet; PPVT-5, Peabody picture vocabulary test; EVT-3, expressive vocabulary test.

mv-ASD participants had prior ASD diagnosis that was confirmed by expert clinicians and licensed psychologists at the Lurie Center for Autism, using the DSM-5 (DSM-5; American Psychiatric Association, 2022) symptom checklist and Social Communication Questionnaire (SCQ; Rutter et al., 2003). NT participants did not meet criteria for ASD based on the DSM-5 symptom checklist and did not have a family history of ASD in any first-degree relatives based on self-report. Minimally verbal status within the mv-ASD group was determined using the following criteria: (1) spoken language repertoire of less than 30 words or word approximations used meaningfully, based on parental report; and/or (2) Expressive Vocabulary Test (EVT-3) scores ≤1st percentile. We refer to as ‘non-speaking’ a few participants whose words, while not full productions, consisted of recognizable approximations of the target words consistent with family report, thus allowing analysis with our metrics. Parents of participants with mv-ASD were also given the MacArthur-Bates Communication Development Inventories to complete, though these were secondary for an expressive word count as they were not always consistently completed or returned.

Using an intake questionnaire with parents, a review of participants’ medical records and medical history was undertaken at the time of screening participants to ensure eligibility for the study. All participants had normal or corrected to normal hearing and vision, and English as the primary language used at home. The presence of various comorbid disorders was excluded across both groups, including those that are known to affect speech such as schizophrenia, bipolar disorder, severe major depressive disorder, severe anxiety disorder, substance use disorder, neurological problems (e.g., cerebral palsy, lifelong encephalopathy, in utero stroke, severe anoxia at birth, meningitis), and voice pathologies (e.g., polyp or node on vocal folds).

### Protocol

Eligible participants completed 3 in-person visits at the Lurie Center for Autism as part of a broader multi-modal study involving speech, ocular, and handwriting skills. The first visit entailed intake screening and standardized clinical testing consisting of a battery of neuropsychological assessments of all participants and the other two visits were dedicated to the experimental protocol. In the third visit, four speech tasks, diadochokinetic sequencing (DDK; Kent et al., 2021; Modolo et al., 2011) and single-word Imitation, Naming, and Reading were administered while audio was recorded. The 2nd visit included non-speech components of the larger protocol (ocular and hand dexterity). All procedures were approved by the Massachusetts General Brigham Institutional Review Board (protocol P000526).

*Experimental speech tasks:* Participants completed three trials of DDK, which included repeating the syllables ‘pa’, ‘ta’, and ‘ka’ separately for 10 s and the syllable sequence ‘pa-ta-ka’ for 10 s, and three trials of single-word Imitation, Naming, and Reading tasks which included a list of 12 target words, with 6 core words - “book,” “dog,” “girl,” “kitten,” “apple,” “potato” - and 6 familiar words selected by parents/caregivers of mv-ASD adults from a list of commonly-used words such as “cup,” “ball,” and “book.” NTs also produced all 12 target words. For the Imitation task, the participant was asked to verbally repeat a target word spoken by the examiner, for the Naming task to verbalize the target word when shown a picture, and for the Reading task to read a printed target word. The tasks were chosen to introduce increasing levels of cognitive demand in speaking. All tasks were repeated 3 times each and in the same order (DDK, Imitation, Naming, and Reading) for all participants. Production of DDK and target words, or attempted approximations of target words for those who were non-speaking, were included in analyses.

An approach to effectively elicit speech in our mv-ASD cohort was essential. The words were selected by parents, were familiar to all subjects, and were produced under the three above conditions: Imitation, Naming and Reading. The tasks were modeled by the experimenter, when needed, to facilitate task comprehension and compliance as is frequently done in research and in the clinic when working with difficult-to-test populations. This was straightforward to achieve in the Imitation task, as the participants had to repeat the words produced by the experimenter one at a time. For Naming and Reading, the tasks were modeled by the experimenter using a non-target word (that is, a word not included in the protocol list) but also familiar to the participants to demonstrate the elicitation of the target words when presented under the same conditions.

The audio was collected as part of a broader multi-modal study involving speech, ocular, and hand skills. Specifically, a Microsoft Surface Book 2 (Microsoft Corp., Redmond, WA) was placed in front of the participant. A Sennheiser MKE 600 Shotgun Microphone (Sennheiser, Germany), connected to the Surface using a Rubix 44 (Roland Corporation, Japan), collected audio at a sampling rate of 48,000 Hz, while a Tobii Nano Pro (Tobii, Sweden) eye tracking strip collected eye movements on the screen at 60 Hz. Each DDK syllable sequence and each word in the single-word task was time stamped and segmented for participant speech using the Praat software. The speech and eye protocols were presented to the child using custom software built in MATLAB (Mathworks, Natick, MA) utilizing Psychtoolbox (Kleiner et al., 2007). We focused on development of an off-body platform to include mv-ASD individuals with high tactile sensitivity (Riquelme et al., 2016). This customized off-body system was used to collect data on all participants of both the mv-ASD and NT cohorts.

*Neuropsychological Assessments:* Participants also completed a comprehensive battery of standardized neuropsychological assessments that measured their fine motor, cognitive, and language abilities (Table 1). Two assessments were used to measure fine motor skills: (1) The Grooved Pegboard Test (GPT), which requires participants to place grooved pegs into holes on a board as quickly and accurately as possible using their dominant hand, and measures the dexterity and visuomotor coordination of the hands and fingers (Mathews and Klove, 1964); and (2) the Beery-Buktenica Developmental Test of Visual Motor Integration, Sixth Edition (VMI-6), which involves copying a series of geometric shapes and measures the ability to integrate visual information with the fine motor action of drawing (Beery et al., 2010). The Stanford-Binet Intelligence Scales, Fifth Edition (SB-5) - Abbreviated Battery was used to capture verbal and non-verbal cognitive skills (fluid reasoning, knowledge; Roid, 2003). However, only nonverbal fluid reasoning subtest scores were used in analyses, as all mv-ASD participants were at floor on the verbal subtest. For language abilities, we used the Expressive Vocabulary Test, Third Edition (EVT-3), a measure of expressive vocabulary skills, that requires participants to provide a single-word verbal response to questions about pictures (i.e., labeling an object or providing the number of objects shown; Williams, 2018). For receptive language skills, the ability to understand the meaning of spoken words was measured using the Peabody Picture Vocabulary Test, Fifth Edition (PPVT-5) where participants identify which picture, out of 4 possible options, matches a spoken word (Dunn, 2018). All assessments were administered by a licensed clinical psychologist, or trained postdoctoral fellow or research coordinator.

### Analysis of speech features

*Feature foundation*: For each task (DDK, Imitation, Naming, and Reading), this study primarily focused on 3 base features, the first 3 speech formants, pitch, and envelope, and secondarily on 1 alternate feature for each of the laryngeal and articulatory systems (Figure 1). The latter two features were chosen for their standard use and effectiveness in the detection of a variety of neurological disorders (Cummins et al., 2015; Low et al., 2020). Characterization of motor dynamics associated with each subsystem was derived from these features using correlation measures (Hadd and Rodgers, 2021).

Representing the articulatory subsystem, 3 formant (formants F1-F3) time trajectories, i.e., vocal tract resonances over time, were extracted as one base feature set using the Kalman-based open-source autoregressive moving average software (KARMA; Mehta et al., 2012). Mel-frequency cepstral-coefficients (MFCCs), representing vocal tract spectral shape over time in 12 spectral (mel) bands (Davis and Mermelstein, 1980; Quatieri, 2001), were also extracted using Praat software’s built-in functionality (Boersma and Weenink, 2018). To represent the laryngeal subsystem, fundamental frequency F0 (pitch), representing vocal fold rate of vibration, was extracted as a base feature using the built-in module of Praat (Boersma, 1993; Boersma and Weenink, 2018) followed by post-processing. The default Praat pitch floor and ceiling are 75 Hz and 600 Hz. But because we are working with adult speech and to avoid pitch-doubling artifacts, we chose a ceiling of 250 Hz. Additionally, in computing pitch velocity, we appled a threshold of 8 Hz to remove changes in pitch across successive frames that are > 8 Hz or < −8 Hz. Our secondary laryngeal feature, cepstral peak prominence (CPP), representing vocal fold vibration stability, was extracted utilizing a custom-built MATLAB application based on existing techniques (Awan et al., 2010; Fraile and Godino-Llorente, 2014; Heman-Ackah et al., 2002; Hillenbrand et al., 1994).

To represent the respiratory subsystem, a speech envelope was extracted using a custom MATLAB application that provides a smooth contour of amplitude peaks of a waveform based on an iterative time-domain signal envelope estimation that applies low-pass filtering and peak re-insertion (Horwitz-Martin et al., 2016; Röbel and Rodet, 2005). Unlike a standard envelope such as AM demodulation that low-pass filters the signal absolute value, our approach results in tracking peaks in the signal, e.g., periodic vocal tract responses during voicing and peaks in consonants, while providing a smooth function. We emphasize, however, that it is not known if atypical change in the envelope derives from lack of control of subglottal pressure (respiratory) or lack of control of closure and release of the lips or tongue (articulatory) or a laryngeal influence (see Discussion section). Therefore, we use the term ‘respiratory’ loosely to be associated with any temporal variation in air pressure that results in a change of signal amplitude. Finally, in addition, we investigated the velocities of each measure as estimated by the first central difference of a feature trajectory.

The base and alternate feature extraction methods involved frame-based analysis over a sliding time window that results in time-series trajectories of features. Specific durations of the time window were based on default parameters for each feature extraction algorithm given in the literature (in the range 20–30 ms). All acoustic feature trajectories were obtained at (a frame rate) 100 Hz, except for pitch, which was extracted at 1000 Hz, and MFCCs, which were extracted at 200 Hz. Specifics of each algorithm can be found in the above references.

**Figure 3:**
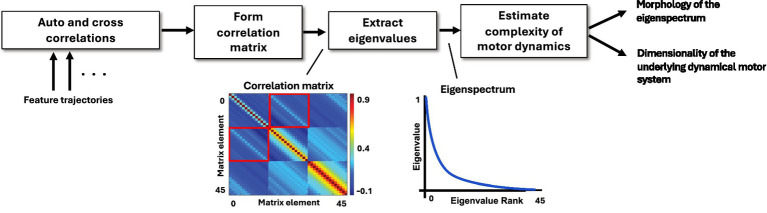
Pipeline for feature extraction and complexity assessment. For each subsystem and task, we explore the nature of complexity through eigenspectra morphology and dimensionality of the embedding space. As illustration, a correlation matrix and eigenspectrum corresponding to 15 auto- and cross-correlations of 3 feature trajectories is shown.

*Complexity of speech motor dynamics:* We compared dynamics of speech performance across mv-ASD and NT groups using the above acoustic feature trajectories as proxies for subsystem motor movements. Correlation-based measures of complexity of various aspects of motor dynamics were derived for each production subsystem (Quatieri et al., 2017; Talkar et al., 2024b). The pipeline for complexity assessment is illustrated in Figure 3. For envelope, pitch, and CPP features, we computed the auto-correlation of single temporal trajectories, while for formant and MFCC features, the auto- and cross-correlations of multiple temporal trajectories.

**Figure 4:**
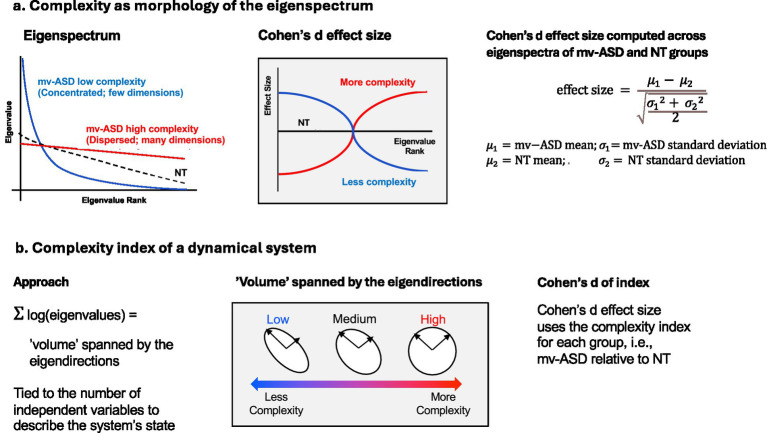
Perspectives on complexity of movement dynamics. **(a)** Left panel: concentrated or dispersed eigenspectrum reflects the number of modes of a dynamical system and hence the degree of *complexity*, i.e., low (blue) or high (red) complexity associated with few or many dimensions, respectively; the dashed black line represents the NT ‘healthy sweet spot’ that falls between mv-ASD cases with low or high complexity. Each function shape (exponential or linear) is intended to schematize different eigenspectral dropoffs, reflecting different dimensions, and not associated with a deeper interpretation. The narrow exponential implies a highly concentrated curve corresponding to a small set of eigenvalues (dimensions) that govern the dynamics, the nearly flat curve many dimensions, and the intermediary sweet spot. **(a)** Middle and right panels: the complexity of a mv-ASD group relative to a NT group can be measured through the Cohen’s d effect size. A positive-to-negative effect size reflects an ASD group with lower complexity while negative-to-positive higher complexity. **(b)** Volume of space (concentrated or dispersed) spanned by eigendirections. Σ log(eigenvalues) provides a complexity index, i.e., a single variable, and is tied to the number of independent dimensions needed to describe the underlying dynamical system state. Graphics adapted from (Talkar, 2023; Copyright Tanya Talkar).

Measures of motor complexity (Figure 4a) were extracted through the construction of correlation matrices, using correlations of feature trajectories, capturing the relative coupling of the time series at multiple time delays. Eigenvalues extracted from these correlation matrices represent the number of dimensions, i.e., independent degrees of freedom, required to fully describe the time-delay embedding space of the time series. If there are a few large eigenvalues and many smaller eigenvalues, this represents lower complexity. Four time-delay scales, i.e., time intervals in sampling the correlation functions, were utilized for the speech analysis: 10 ms, 30 ms, 70 ms, and 150 ms. These values aim to capture temporal resolution of dynamics at different time scales such as associated with phoneme, syllable, bi-syllable and word-level sound components. More information about the generation of correlation-based features is laid out in studies of Major Depressive Disorder (Williamson et al., 2019; Quatieri et al., 2017) and Autism Spectrum Disorder (Talkar, 2023; Talkar et al., 2024a; Talkar et al., 2024b).

The Cohen’s d effect sizes across the eigenspectrum of an ASD group relative to a NT group determines the relative complexity of the underlying dynamical systems (middle and right panels of Figure 4a). A pattern of positive-to-negative effect size indicates that the ASD group has lower complexity of signals as compared to the NT group, while a pattern of negative-to-positive effect size indicates higher complexity in the ASD group. If the effect sizes are small across all eigenvalues when comparing the groups, the underlying signals of the two groups have similar levels of complexity. When comparing the mv-ASD group to the NT group, higher complexity means more variability and perhaps more ‘erratic’ motor control; while less complexity means more coupled movements, i.e., a more constrained dynamical system.

Although the morphology of the effect-size functions sheds light on the relative nature of the complexity, given the multidimensional nature of the eigenspectrum, a single complexity index (Figure 4b) was also determined, thus allowing a more succinct representation. The approach we chose involves the sum of the log of the eigenspectral values which provides a measure of the tightness of the spatial ‘volume’ (associated with independent modes) of the underlying dynamics of the motor system. A flat space has few dimensions and small volume. Toward reducing sensitivity to positive or negative low-energy noisy eigenvalues, we did not include those of high rank (> 2/3 max eigenvalue rank) and used the real log component.

To expand on this interpretation of a complexity index based on a volume concept, we call on some standard linear algebra. For a correlation matrix, the log(determinant of the matrix) is the log of the product of the eigenvalues or the sum of the log of the eigenvalues. The geometric interpretation of the determinant is that its absolute value represents the ‘scatter volume’ of the signal space represented by the eigenvalues and corresponding eigenvectors. As illustrated in the middle panel of Figure 4b, a flat volume has few dimensions, a round volume many dimensions, and an oval volume corresponds to a ‘healthy sweet spot’. Various standard linear algebra references cover this volume concept (e.g., Margalit and Rabinoff, 2019).

An alternative to this geometric view is a physical analogy to motoric actions in speech production (e.g., the tongue, lips, and jaw in articulation). Here we compare against states of finger dexterity (Fetters, 2010) where ‘frozen’ fingers move together, excessively coupled and unable to move independently (atypical low dimension), whereas erratically moving fingers cannot synchronize with meaningful movement (atypical high dimension). The ‘healthy sweet spot’ falls between these two states, i.e., the neurotypical dashed line in the top left and horizontal axis in the top middle diagrams of Figure 4a. Interestingly, a similar analogy can be found with gross-motor conditions such as associated with Parkinson’s disease and aging (Vaillancourt and Newell, 2002), supporting the notion of a healthy state being the sweet spot between too little or too much variability. Furthermore, under a condition like Parkinson’s or aging, this state of variability of movement can vary with different body parts and the coordination across body parts, analogous to our speech production subsystem representation.

The resulting acoustic-based complexity measures were also correlated with scores on clinical assessments of nonverbal IQ (NVIQ), expressive and receptive vocabulary (EVT-3 and PPVT-5), and fine-motor skills (VMI-6 and GPT) using Pearson and Spearman correlations.

## Results

### Characterization

In this paper, our focus is on group- and individual-level analyses for our four tasks (DDK, Imitation, Naming, and Reading). Specifically, we address DDK trials combined (pa, ta, ka and pa-ta-ka) and target words combined during each single-word task (Imitation, Naming, Reading) to obtain a holistic sense of the nature of the complexity of movement dynamics. We first highlight group and individual findings on the DDK and single-word Imitation, Naming, and Reading tasks using base acoustic features: vocal tract resonances (formants), vocal fold vibration fundamental frequency (pitch), and lung pressure as indicated by the waveform envelope, as proxies to aspects of the physiology of the articulatory, laryngeal, and respiratory subsystems, respectively. Eigenspectra derived from the correlation structure of feature trajectories reflect movement dynamics of each subsystem. We also investigate the velocity of feature trajectories as a proxy for movement velocity within each subsystem.

**Figure 5:**
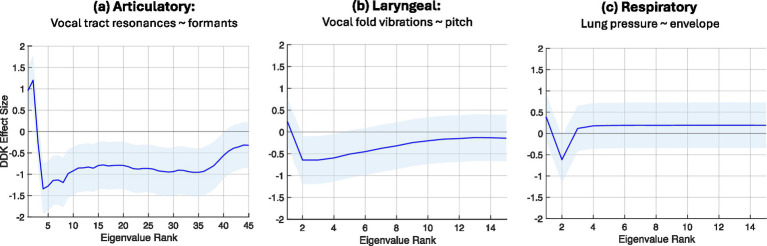
DDK *group findings*: Complexity of motor dynamics for the DDK task based on eigenspectrum effect sizes for the mv-ASD group relative to the NT group averaged over participants in each group. A positive-to-negative effect size reflects a lower complexity of the mv-ASD group, while negative-to-positive reflects higher complexity. If the effect sizes are small across all eigenvalues when comparing the two groups, the groups have similar levels of complexity, and thus a Cohen’s d effect size near zero (the zero horizontal axis). In **(a,b)**, the DDK task reveals in the articulatory and laryngeal systems lower complexity for the mv-ASD group, while in **(c)** the respiratory system effect size is unclear due to its multiple crossing points of the zero horizontal axis. (However, our complexity index in Figure 7 and the distribution spread across all participants in Figure 8c indicate lower group-level mv-ASD complexity.) The 95% confidence interval of each effect size, calculated from the distributions of eigenvalues from the mv-ASD and NT groups is highlighted across the eigenspectra in light blue shading, reflecting a large mv-ASD heterogeneity (see Figure 8 example).

**Figure 6:**
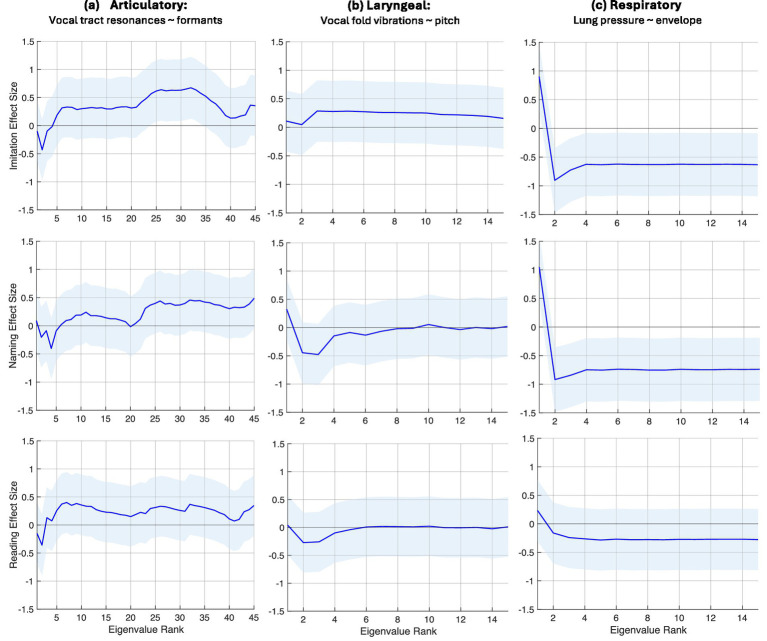
S*ingle-word group findings*: Complexity of motor dynamics for the Imitation, Naming, and Reading single-word tasks based on eigenspectrum effect sizes for the mv-ASD group relative to the NT group averaged over participants in each group and task, reflecting a subsystem- and task-dependence. In **(a)**, the mv-ASD group shows higher complexity in the articulatory system across all word categories. In contrast, **(b,c)** reveal lower complexity for mv-ASD in laryngeal and respiratory subsystems, except for the imitation task where for the laryngeal subsystem, the effect size suggests higher mv-ASD complexity. The 95% confidence interval of each effect size, calculated from the distributions of eigenvalues from the mv-ASD and NT groups, is highlighted across the eigenspectra in light blue shading reflecting a large mv-ASD heterogeneity.

*Group-level analysis:* For group analysis of each subsystem, we first computed Cohen’s d effect sizes of the average eigenspectra for each task across our two groups, i.e., the mv-ASD relative to the NT group. Eigenspectra were averaged over participants in each group (27 mv-ASDs and 27 NTs) and across all components of each task, i.e., all components of the DDK task and all words in each single-word task (Imitation, Naming, and Reading). Effect sizes were computed for each feature trajectory associated with the 3 speech subsystems. Figures 5, 6 illustrate these effect sizes for the DDK and single-word tasks, respectively.

**Figure 7:**
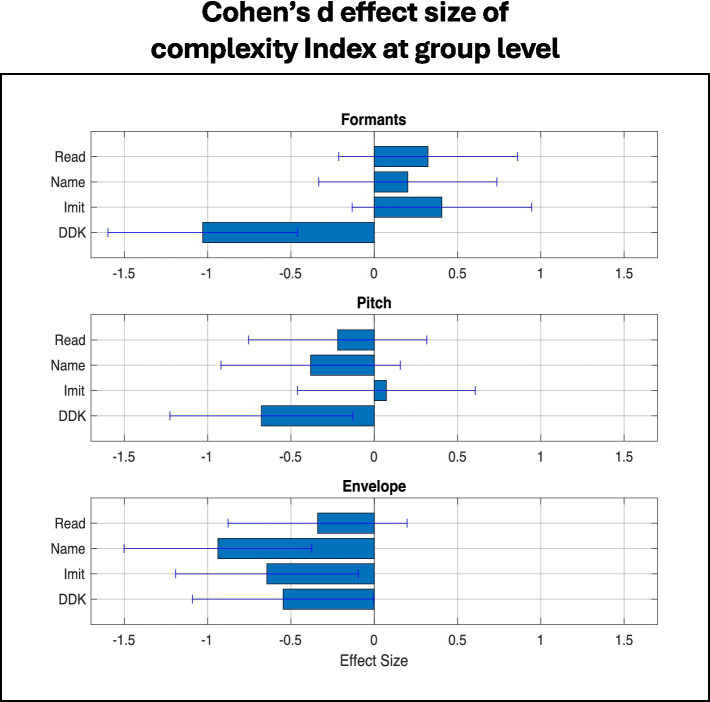
Complexity index effect sizes of the mv-ASD group relative to the NT group for DDK and single-word tasks for each base feature. The Cohen’s d effect sizes of the mv-ASD relative to the NT group reveal moderate-to-large effect sizes in most cases. Complexity index effect sizes follow our observations on relative eigenspectrum morphology (Figures 5, 6) except for the respiratory subsystem under the DDK task. Cohen’s d effect size for each subsystem and task is shown with a 95% confidence interval, calculated from the distributions of effect sizes (Hedges and Olkin, 1985; Goulet-Pelletier and Cousineau, 2018), reflecting the large mv-ASD heterogeneity (see Figure 8 example).

**Figure 8:**
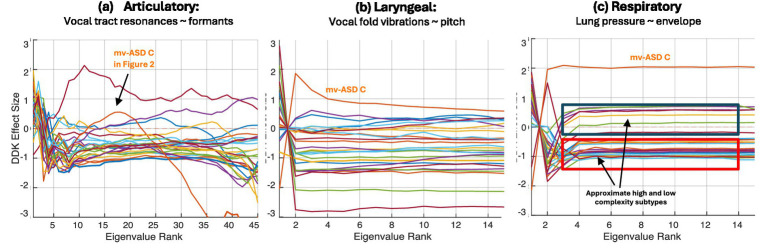
*Individual findings*: Complexity of motor dynamics for the DDK task for mv-ASD participants in **(a)** articulatory, **(b)** laryngeal, and **(c)** respiratory subsystems. Effect size for each mv-ASD shown relative to NT comparison with reference as the average eigenspectrum across all NTs (Each color denotes a different participant.); mv-ASD C (non-speaking) is highlighted as an outlier, having large variability in production within and across words.

A striking observation across the two figures is the consistency of the nature of the eigenspectra (on average) across the DDK and three single-word tasks for each subsystem, but reflecting some subsystem-dependence. Via the morphology of the eigenspectral effect size for the DDK task, in Figures 5a,b mv-ASD shows lower complexity (i.e., more coupled system dynamics) in the articulatory and laryngeal subsystems while the eigenspectral effect size for the respiratory system in Figure 5c is unclear due to its multiple crossing points of the zero horizontal axis. (However, our complexity index to be discussed in Figure 7 and the distribution spread across all participants in Figure 8c indicate lower group-level mv-ASD complexity.) For the single-word tasks, mv-ASD show higher complexity in the articulatory system (i.e., more variable system dynamics) in Figure 6a but lower complexity in laryngeal and respiratory subsystems in Figures 6b,c, except for the imitation task where for the laryngeal subsystem, the effect size suggests higher mv-ASD complexity. For all task categories, the 95% confidence interval of each effect size (Hedges and Olkin, 1985; Goulet-Pelletier and Cousineau, 2018), calculated from the distributions of eigenvalues from the mv-ASD and NT groups, is highlighted across the eigenspectra in light blue shading, reflecting a large mv-ASD heterogeneity (see Figure 8 example).

Our complexity index defined in the Methods section was also computed for each of the 3 subsystems (articulatory, laryngeal, respiratory) for the DDK task and each single-word task (Imitation, Naming, Reading). The Cohen’s d of the index of the mv-ASD relative to the NT group (Figure 7) indicates moderate-to-large effects size across most cases. Specifically, for all three subsystems, we observe a lower complexity in the mv-ASD group for the DDK task. For the single-word tasks, on the other hand, we see higher articulatory complexity for Imitation, Naming, and Reading, while a lower laryngeal and respiratory complexity for the single-word tasks, except for the laryngeal subsystem with the Imitation task that suggests a near-zero index difference thus indicating close to NT in this category.

We observed earlier the large spread of eigenspectra about the average; likewise, this is seen in the single-variable Cohen’s d effect size via its 95% confidence interval (CI) in Figure 7, calculated from the distributions of effect sizes (Hedges and Olkin, 1985; Goulet-Pelletier and Cousineau, 2018), reflecting the large mv-ASD heterogeneity (see Figure 8 example). The spread of the confidence intervals of the effect sizes tends to be consistent with those of the eigenspectra in Figures 5, 6.

Characteristics of the Cohen’s d effect sizes follow our observations on eigenspectrum morphology (Figures 5, 6) except for the DDK/respiratory case. This discrepancy may be explained by noting that deducing complexity from the eigenspectrum morphology is qualitative, while the log operation in computing the index provides a quantitative metric based on the concept of ‘volume.’ Furthermore, the log operation tends to add weight to the high-rank low-energy eigenvalues.

Less complexity can correspond to less variability due to less high frequency contribution in the temporal feature trajectory. This has been shown by simulation in the work of Talkar (2023). Correspondingly, it is interesting to observe that lower complexity of movement dynamics in the laryngeal and respiratory systems at a group level may be associated with a ‘monotonous’ or ‘robotic’ voice historically attributed to the ASD population (Franich et al., 2021; Kissine and Geelhand, 2019; Shriberg et al., 2001) where monotone pitch for example has little high-frequency variation. This depiction, however, does not hold for all individuals with ASD, as seen in the CI spread of Figure 7; some ASD individuals are described as having more of a ‘sing-song voice’ or more variable voice (Fusaroli et al., 2017) that may correspond to higher complexity due to a high-frequency contribution (Talkar, 2023). How complexity changes with specific signal contributions has been explored further in Talkar (2023) and remains an important research area in mv-ASD vocalizations.

**Figure 9:**
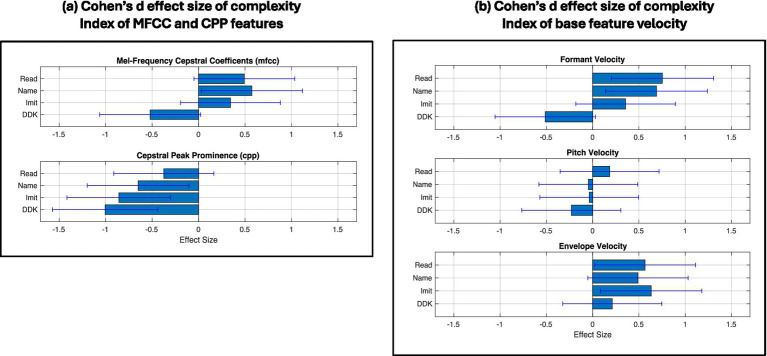
Cohen’s d effect size complexity index of mv-ASD group relative to NT group for two categories of alternate features: **(a)** Popular MFCC representing the articulatory subsystem and CPP representing the laryngeal subsystem; **(b)** Velocity of base features (formants, pitch, and envelope). Cohen’s d effect size for each subsystem and task is shown with a 95% confidence interval, calculated from the distributions of effect sizes (Hedges and Olkin, 1985; Goulet-Pelletier and Cousineau, 2018), reflecting the large mv-ASD heterogeneity (Figure 8 example).

As noted earlier, we considered two other feature sets: MFCC and CPP values for articulatory and laryngeal subsystems, respectively, as well as the velocity of base features. In comparing Cohen’s d effect sizes of MFCCs and CPPs (Figure 9a) with those of our base formant and pitch features (Figure 7), we find for the articulatory subsystem that effect sizes in MFCC are slightly larger in Reading and Naming conditions, but smaller in imitation and much smaller in DDK. On the other hand, effect sizes for CPP are uniformly larger than those of our base pitch features.

Figure 9b shows that Cohen’s d effect size for envelope velocity is positive in contrast to negative for envelope magnitude (as seen in the effect sizes of Figure 7) indicating more variability in how fast loudness changes. This is consistent with a more ‘bursty’ signal especially as we sometimes observed in word and syllable onsets in our dataset. Table 2 summarizes a discrete perspective of the relative complexity measures based on Figures 5–9.

**Table 2:** Summary of increased (high) vs. decreased (low) complexity in mv-ASD relative to NT group as a function of task and subsystem feature based on the effect sizes in Figures 5–9.

Task	Articulatory (base)		(alternate)	Laryngeal (base)		(alternate)	Respiratory (base)	
	Formant	(vel)	MFCC	Pitch	(vel)	CPP	Envelope	(vel)
Reading	high	high	high	------	high	low	low	high
Naming	high	high	high	low	-----	low	low	high
Imitation	high	high	high	------	-----	low	low	high
DDK	low	low	low	low	low	low	low	high

------- denotes an absolute effect size less than 0.20. (vel) denotes base feature velocity.

*Subject-level analysis:* Though we observed in the above analysis, often distinct consistencies in eignenspectrum morphology across tasks and/or subsystems, we observed subject-dependence in the DDK and the single-word tasks. Examples of this variability for the three base speech features (formants, pitch, and envelope) with the DDK task are shown in Figure 8, reflecting the heterogeneity in the mv-ASD group. Each effect size was derived as a Cohen’s d for each mv-ASD participant eigenspectrum against the averaged NT eigenspectra. We observed a variability with respect to this average NT eigenspectrum, ranging from low-to-high complexity, with the laryngeal subsystem manifesting the largest variation for this case. Some profiles suggest potential for creating subtypes using a complexity metric. Similar eigenspectral variations across participants were observed for the single-word tasks, consistent with the confidence intervals in Figure 6. We note that certain outliers such as ASD C (example in Figure 2) are representative of a few mv-ASD participants in our dataset who are non-speaking, resulting in large deviation in complexity from the group mean. As noted earlier, and in Figure 8, our aim is to show the subject dependence in the DDK and single-word production tasks, highlighting differences within mv-ASD but as well the distinction of non-speaking ASD, and the potential to use our complexity metric to compare words and word approximations even across a reduced range of verbal ability in ASD.

### Correlation with clinical assessment

In investigating associations with clinical assessments, we correlated our acoustic features with scores on NVIQ, EVT-3, PPVT-5, VMI-6 and GPT (dominant hand). Our initial analyses included both mv-ASD and NT groups together. In addition to Pearson correlations, Spearman correlations were computed due to the presence of outliers (e.g., non-speaking participants) and flexibility in not restricting the data to be Gaussian (Normal) and feature-assessment relations to be linear. While the Spearman is more robust to outliers due to use of rank, Pearson is more sensitive due to its use of value and thus potentially more accurate, thus providing greater insight than either alone. For both correlation approaches, we applied the Bonferroni correction assuming a 5-category comparison because our key objective in this part of the study is correlation analysis against 5 clinical assessments. Thus in our correlation tables, we have marked correlations whose adjusted *p*-values remain under 0.05 after multiplying by a factor 5.

Given the multidimensional nature of our eigenspectrum representation of complexity, as described above, a single complexity index was introduced. This index was computed for each of the 3 subsystems (articulatory, laryngeal, and respiratory) for each single-word task (Imitation, Naming, Reading) as well as the DDK task. Highlighting a few key results, we see in Table 3 that at the group level, i.e., combined mv-ASD and NT cohorts, articulatory features (formants) were most consistent in correlating with expressive vocabulary (EVT-3 scores) across all tasks, while the DDK task was most consistent in correlating across all subsystems and assessment scores.

**Table 3:** Pearson and Spearman correlations of the complexity index with clinical assessment scores across both groups (mv-ASD and NT).

Assessment	Tasks	Articulatory (formants)	Laryngeal (pitch)	Respiratory (envelope)
EVT (R, P)	DDK Imitation Naming Reading	(0.38, 0.007)* (0.47, 0.000)* (−0.39, 0.005) (−0.24, 0.096) **(−0.28, 0.050) (−0.30, 0.034)** **(−0.35, 0.011) (−0.35, 0.011)**	(0.27, 0.056) (0.43, 0.002)* ----------------- ----------------- (0.24, 0.087) (0.36, 0.007)* ----------------- -----------------	(0.33, 0.020) (0.46. 0.000)* ------------------ ---------------- (**0.32, 0.023) (0.35, 0.013)** ------------------ ----------------
PPVT (R, P)	DDK Imitation Naming Reading	(0.43, 0.002)* (0.54, 0.000)* ------------------ ---------------- ------------------ ---------------- (−0.24, 0.093). **(−0.36, 0.012)**	(0.42, 0.003)* (0.55, 0.000)* ----------------- ----------------- (0.29, 0.044) (0.44, 0.002)* ----------------- (0.26, 0.076)	(0.43, 0.002)* (0.54, 0.000)* (0.30. 0.037) ----------------- (0.40, 0.004)* (0.38, 0.007)* ----------------- -----------------
NVIQ (R, P)	DDK Imitation Naming Reading	0.48, 0.003)* (0.53, 0.000)* (−0.26, 0.062) ----------------- ------------------ ----------------- ------------------ -----------------	(0.23, 0.095) (0.39, 0.003)* ------------------ ----------------- ------------------ (0.25, 0.071) ------------------ -----------------	(0.31, 0.025) (0.43, 0.001)* ------------------ ----------------- **(0.25, 0.060) (0.29, 0.034)** ------------------ -----------------
VMI (R, P)	DDK Imitation Naming Reading	(0.42, 0.002)* (0.45, 0.000)* ------------------ ----------------- (−0.25, 0.064) ----------------- ------------------ (−0.27, 0.053)	(0.28, 0.040) (0.38, 0.004)* ----------------- ----------------- --------------------- (0.26.0.058) ----------------- ------------------	**(0.31, 0.02**) (0.40, 0.003)* ----------------- ----------------- (0.34, 0.013) (0.38, 0.004* ------------------ -----------------
GPT (R, P) dominant	DDK Imitation Naming Reading	**(−0.43, 0.002**)* (−0.48, 0.000)* (0.31, 0.023) -------------------- ------------------ ---------------- ------------------ ----------------	(−0.27, 0.052) (−0.40, 0.003)* ------------------- ---------------- ------------------- ---------------- ------------------- ----------------	(−0.28, 0.040) (−0.40, 0.003)* ------------------- ---------------- (−0.30, 0.026) (−0.43, 0.001)* ------------------- ------------------

Base speech features (formants, pitch, envelope) represent the 3 subsystems, respectively. Within each subsystem column, Pearson and Spearman correlations are left and right location respectively, along with their *p* values. Bold denotes correlations with *p* ≤ 0.05; unbold: with 0.05 < *p* ≤ 0.10; and dashed line (−-----------) with *p* > 0.10. Correlations marked with * survive a 5-factor Bonferroni adjustment (R, correlation; P, *p*-value significance).

**Figure 10:**
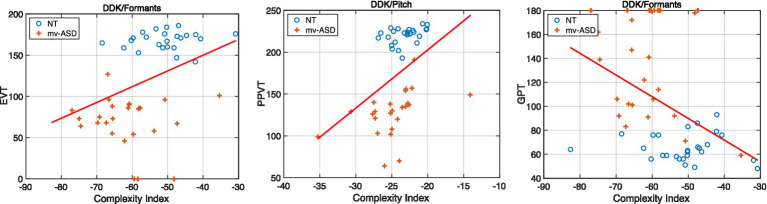
Example scatter plots of high-correlating cases from Table 3, illustrating the relation of our complexity index for selected subsystem features and speech tasks against various clinical assessments. Blue circles denote NT while red crosses mv-ASD. Plots clockwise: formants (articulatory) from the DDK task for the EVT assessment, pitch (laryngeal) from the DDK task for the PPVT assessment, and formants (articulatory) from the DDK task for the GPT assessment. Group Pearson/Spearman correlations (mv-ASD and NT together) from Table 3 are, respectively: (0.38, 0.007)/(0.47, 0.000), (0.42, 0.003)/(0.55, 0.000), and (−0.43, 0.002)/(−0.48, 0.000). Linear polynomial fits (red solid lines) reveal trends consistent with the Pearson values of Table 3 and give insights on the influence of outliers.

Example scatter plots are shown in Figure 10, illustrating the relation of our complexity index for selected subsystem features and speech tasks against various clinical assessments. Observations include: (1) a shift-left of the mv-ASD complexity index/assessment distribution (i.e., lower complexity) relative to that of NT participants, consistent with effect sizes reported in Figure 7 and (2) in the index-assessment space, a tighter or narrower clustering of the NT distributions relative to the more dispersed mv-ASD distributions. Another noteworthy observation involves the presence of outliers, i.e., points off the general trend due to for example non-speaking individuals. For the three cases of Figure 10 (cf. Table 3; Figure 10 caption), applying the Spearman correlation has increased the correlation, likely due to its relative insensitivity to outliers.

In contrast to using both mv-ASD and NT groups together, Table 4 shows correlations with clinical assessments for the mv-ASD group-only. Articulation Pearson correlations remain high (and even higher) for the single-word tasks. Articulatory complexity for DDK, however, no longer correlates with EVT-3. Furthermore, there are few other correlation values that meet the significance threshold. Results with the mv-ASD group analysis for Spearman correlations manifest in Table 4 as mixed. Table 5 gives the companion NT group-only correlation results. With both the within-mv-ASD and within-NT tables, we generally observe a sparcity of correlations with p-values less than 0.05. The highest correlating ones are clustered for EVT and PPVT perhaps due to our speech motor measures being most directly related to these language assessments. In both cases, Spearman correlations often strengthen the Pearson (likely due to the presence of outliers), sometimes weaken the correlation as in the articulatory case, or show little change.

**Table 4:** Correlations of complexity index with clinical assessment scores within the mv-ASD group only.

Assessment	Tasks	Articulatory (formants)	Laryngeal (pitch)	Respiratory (envelope)
EVT (R, P)	DDK Imitation Naming Reading	------------------ ---------------- (−0.51, 0.008)* ---------------- **(−0.44, 0.024)** ---------------- (−0.60, 0.001)* -----------------	---------------- (0.43, 0.002)* ---------------- ----------------- ---------------- (0.36, 0.007)* ----------------- -----------------	**(−0.44. 0.025)** (−0.35, 0.084) (−0.39, 0.091) **(−0.41, 0.037)** ------------------ ----------------- ------------------ -----------------
PPVT (R, P)	DDK Imitation Naming Reading	------------------ ------------------ ------------------ ------------------ ------------------ ------------------ ------------------ ------------------	(0.42, 0.044) (0.61, 0.001)* (0.37, 0.075) (0.56, 0.004)* ----------------- (0.40, 0.055) ----------------- -----------------	----------------- ----------------- ----------------- ----------------- ----------------- ----------------- ----------------- -----------------
NVIQ (R, P)	DDK Imitation Naming Reading	----------------- ----------------- ----------------- ----------------- ----------------- ----------------- ----------------- -----------------	----------------- ----------------- ----------------- ----------------- ----------------- ----------------- ----------------- -----------------	**(−0.45, 0.019) (−0.42, 0.028)** ------------------ ----------------- ------------------ ---------------- ------------------ -----------------
VMI (R, P)	DDK Imitation Naming Reading	------------------ ----------------- ------------------ ----------------- ------------------ ----------------- ------------------ -----------------	----------------- ----------------- ----------------- ----------------- ----------------- ----------------- ----------------- -----------------	(−0.42, 0.031) (−0.51, 0.007)* (−0.33, 0.095) **(−0.45 0.018)** ------------------ ----------------- ------------------ -----------------
GPT (R, P) dominant	DDK Imitation Naming Reading	----------------- ----------------- ----------------- ----------------- ----------------- ----------------- ----------------- -----------------	----------------- ----------------- ----------------- ----------------- ----------------- ----------------- ----------------- -----------------	----------------- ----------------- ----------------- ----------------- ----------------- ----------------- ----------------- -----------------

Base speech features (formants, pitch, envelope) represent the 3 subsystems, respectively. Within each subsystem column, Pearson and Spearman correlations are left and, right respectively, along with their *p* values. Bold denotes correlations with p ≤ 0.05; unbold: with 0.05 < p ≤ 0.10; and dashed line (−--------) with *p* > 0.10. Correlations marked with * survive a 5-factor Bonferroni adjustment (R, correlation; P, *p*-value significance).

**Table 5:** Correlations of complexity index with clinical assessment scores within the NT group only.

Assessment	Tasks	Articulatory (formants)	Laryngeal (pitch)	Respiratory (envelope)
EVT (R, P)	DDK Imitation Naming Reading	----------------- ----------------- ----------------- ------------------ (−0.34, 0.092) **(−0.38, 0.059)** (**−**0.35, 0.089) **(−0.44, 0.026)**	(0.40, 0.051) **(0.40, 0.045)** ----------------- ----------------- (0.44, 0.028) (0.58, 0.002)* ----------------- -----------------	----------------- ----------------- ----------------- ----------------- ----------------- ----------------- **(0.46, 0.021)** (0.37, 0.065)
PPVT (R, P)	DDK Imitation Naming Reading	------------------ ------------------ ------------------ ------------------ ------------------ **------------------** ------------------ **(−0.43, 0.034)**	(0.39, 0.055) **(0.44, 0.031)** ----------------- -----------------(0.43, 0.032) (0.71, 0.000)* (0.36, 0.075) (0.39, 0.051)	----------------- ----------------- ----------------- ----------------- ----------------- ----------------- **(0.42, 0.036)** -------------------
NVIQ (R, P)	DDK Imitation Naming Reading	(0.34, 0.085) --------------------- ----------------- ----------------- ----------------- ----------------- ----------------- -----------------	----------------- ----------------- ----------------- ----------------- ----------------- ----------------- (0.33, 0.091) --------------------	------------------ ----------------- ------------------ ----------------- ------------------ ---------------- ------------------ -----------------
VMI (R, P)	DDK Imitation Naming Reading	------------------ ----------------- ------------------ ----------------- ------------------ ----------------- ------------------ -----------------	----------------- ----------------- ----------------- ----------------- ----------------- ----------------- ----------------- -----------------	------------------ ----------------- ------------------ ----------------- ------------------ ----------------- ------------------ -----------------
GPT (R, P) Dominant	DDK Imitation Naming Reading	----------------- ----------------- ----------------- ----------------- ----------------- ----------------- ----------------- -----------------	----------------- ----------------- (0.52, 0.005)* (0.51, 0.006)* ----------------- ----------------- ----------------- -----------------	----------------- ----------------- ----------------- ----------------- (−**0.38, 0.048)** (−0.35, 0.078) ----------------- -----------------

Base speech features (formants, pitch, envelope) represent the 3 subsystems, respectively. Within each subsystem column, Pearson and Spearman correlations are left and, right respectively, along with their *p* values. Bold denotes correlations with *p* ≤ 0.05; unbold: with 0.05 < *p* ≤ 0.10; and dashed line (−---------) with *p* > 0.10. Correlations marked with * survive a 5-factor Bonferroni adjustment (R, correlation; P, *p*-value significance).

**Figure 11:**
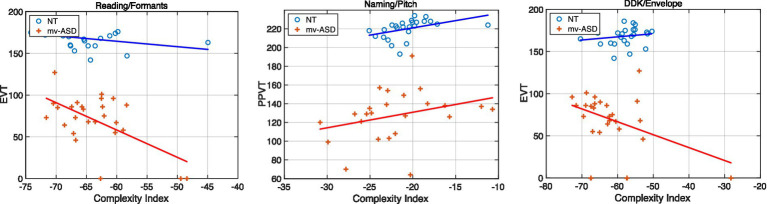
Example scatter plots of high-correlating cases from Tables 4, 5, illustrating the relation of our complexity index for selected subsystem features and speech tasks against various clinical assessments. Blue circles denote NT while red crosses mv-ASD. Plots clockwise: Formants (articulatory) from the Reading task for the EVT assessment, pitch (laryngeal) from the Naming task for the PPVT assessment, and envelope (respiratory) from the DDK task for the EVT assessment. Linear polynomial fits (blue and red solid lines) reveal trends consistent with the Pearson values of Tables 4, 5 and give insights on the effect of outliers. Note from Table 4 that the correlation for the PPVT/Naming/pitch case for mv-ASD nonsignificant according to Pearson but significant according to Spearman. From Table 5, neither of the correlation methods show the EVT/envelope/DDK case to be significant for NT.

Shown in Figure 11 are example scatter plots of high-correlating cases from Tables 4, 5, illustrating the relation of our complexity index for selected subsystem features and speech tasks against language-based clinical assessments, i.e., EVT and PPVT.

Finally, Table 6 shows our highest Pearson and Spearman correlations among alternate features: MFFC and CPP features, and base envelope feature velocity. Envelope velocity for the DDK task provides some of strongest associations (R/*p*-value: 0.84/<0.001 for EVT; 0.87/<0.001 for PPVT) across all assessment scores and features.

**Table 6:** Pearson and Spearman correlations of the complexity index with clinical assessment scores across both groups (mv-ASD and NT) associated with alternate features: MFCC, CPP and envelope velocity, representing articulatory, laryngeal and respiratory subsystems, respectively.

Assessment	Tasks	Articulatory (MFCC)	Laryngeal (CPP)	Respiratory (envelope velocity)
EVT (R, P)	DDK Imitation Naming Reading	(0.36, 0.010)* ------------------ ------------------ ------------------ (−0.27, 0.053) **(−0.29, 0.039)** ------------------ ------------------	**(0.35, 0.013) (0.34, 0.041)** (0.23. 0.108) **(0.28, 0.044)** ----------------- ---------------- ----------------- ----------------	(0.84, 0.000)* (0,69, 0.000)* (−0.36, 0.010*) (−0.36, 0.010)* ------------------- (−0,25, 0,083) (−0.25, 0.078) (−0,27, 0.057)
PPT (R, P)	DDK Imitation Naming Reading	(0.26, 0.077) (0.25, 0.078) ----------------- ---------------- ----------------- ---------------- ----------------- ----------------	(0.51, 0.000)* (0.49, 0.000)* (0.31, 0.031) (0.35, 0.015) **(0.27, 0.046) (0.30, 0.034)** ----------------- -----------------	(0.87, 0.000*) (0.71, 0.000)* (−0.38, 0.007*) (−0.38, 0.007)* (−0.31, 0.030) (−0.27, 0.058) **(−0.35, 0.013) (−0.30, 0.034)**
NVIQ (R, P)	DDK Imitation Naming Reading	(0.35, 0.009)* (0.27, 0.053) ------------------ ------------------ **(−0.28, 0.041) (−0,31, 0.023)** ------------------ ------------------	(0.36, 0.007)* (0.35, 0.011) (0.25, 0.070) (0.26. 0.057) ------------------ ---------------- ------------------ ----------------	(0.76, 0.000)* (0.69, 0.000)* ------------------ (−0.31, 0.022) ------------------ ----------------- (−0.27, 0.054) **(−0.27, 0.046)**
VMI (R, P)	DDK Imitation Naming Reading	(0.37, 0.006)* ----------------- ------------------ (−0.25, 0.065) **(−0.31, 0.025) (−0.39, 0.003)** (−0.25, 0.071) **(−0.28, 0.043)**	(0.40, 0.003)* (0.28, 0.041) **(0.30, 0.033) (0.28, 0.039)** (0.25, 0.075) **(0.27, 0.049)** ----------------- -----------------	(0.80, 0.000)* (0.67, 0.000)* (−0.29, 0.033) (−0.31, 0.021) ------------------ ----------------- **(−0.28, 0.045)** (−0.23, 0.094)
GPT (R, P) Dominant	DDK Imitation Naming Reading	**(−0.28, 0.037) ----------------** (0.25, 0.065) (0.24, 0.079) (0.45, 0.001)* (0.43, 0.001)* (0.35, 0.010) (0.33, 0.016)	(−0.40, 0.003)* (−0.35, 0.010)* (−0.25, 0.067**) (−0.28, 0.042)** ------------------ ----------------- ------------------- ----------------	(−0.72, 0.000)* (−0.64, 0.000)* (0.32, 0.019) (0.33, 0.016) ----------------- ----------------- (0.25, 0.069) (0.25, 0.068)

Within each subsystem column, Pearson and Spearman correlations are left and right respectively, along with their *p* values. Bold denotes correlations with *p* ≤ 0.05; unbold: with 0.05 < *p* ≤ 0.10; and dashed line (−---------) with *p* > 0.10. Correlations marked with * survive a 5-factor Bonferroni adjustment (R, correlation; P, *p*-value significance).

## Summary

Using a first-of-its-kind dataset collected at the MGH Lurie Center for Autism, unlike more standard characterizations, our focus was on movement dynamics of the respiratory, laryngeal and articulatory subsystems representative of motor speech performance in mv-ASD using signal correlation matrices and complexity metrics. We used base features of signal envelope, pitch and formants associated with each speech subsystem, as proxies to underlying movements of the subsystems. We also explored alternate features of MFCC and CPP associated with the articulatory and laryngeal systems, respectively, as well as velocity of base features. These features were examined across four tasks—diadochokinetic sequencing (DDK), and single-word Imitation, Naming, and Reading with a list of 12 words, 6 high frequency core words familiar to all participants and 6 individualized words. With this protocol, we investigated differences in the complexity of subsystem movement dynamics for 27 mv-ASDs versus 27 NTs as a function of task and at both group and individual levels. We looked at complexity qualitatively through the morphology of eigenspectra of correlation matrices associated with movement dynamics of a subsystem but also with a single variable representing the degrees of freedom of each dynamical subsystem. Fewer degrees of freedom reflects less complexity of a system’s movement dynamics.

At a group level, complexity was found to be lower for mv-ASD across all tasks, for the respiratory and laryngeal subsystems, perhaps reflecting a flatter prosody, or a more ‘machine-like’ voice. Articulation, in contrast, was found to have larger complexity for single-word tasks in mv-ASD than NT, reflecting perhaps more variability in speech production. For the MFCC and CPP features, comparisons against base features were mixed: MFCC are slightly larger in reading and naming conditions, but smaller in imitation and much smaller in DDK. On the other hand, effect sizes for CPP are uniformly larger than that for our base pitch features. We also investigated velocity of the three base features (formants, pitch, and envelope velocity), revealing mv-ASD envelope velocity with greater complexity across all tasks which may be associated with a characteristic burstiness in this population. At the mv-ASD individual level, we found a range of feature complexity around the NT group means. This was illustrated by example for the DDK task where we see a spread of eigenspectrum morphologies from lower to higher complexity across the mv-ASD group relative to the NT group. Participants who were most challenged verbally (i.e., non-speaking) stand out as eigenspectrum outliers. Lastly, it is of interest to compare findings from our sEMG work with a subset of the current study’s mv-ASD and NT participants and using the same DDK and single-word tasks of the current study (Zulfikar et al., 2024). Specifically, in this previous work we found that our complexity metric across EMG channel amplitudes from various facial regions (lips, checks, jaw) exhibited typically lower complexity than the NT cohort, associated with greater coupling between movements of these regions and implying fewer degrees of freedom in motor control. In contrast, our complexity measures for the mv-ASD cohort using acoustics as a proxy measure for the articulatory subsystem, the speech subsystem closest to facial dynamics, manifest as higher complexity than their matched NT cohort, except for the DDK task that shows lower complexity.

Using our single complexity index, the resulting acoustic-based features were also correlated with neuropsychological test scores, including non-verbal IQ (NVIQ, from SB-5), expressive and receptive vocabulary (EVT-3 and PPVT-5), and visual-motor integration and pegboard (dominant hand; VMI-6 and GPB) scores. For combined mv-ASD and NT groups, the base articulatory feature (i.e., formants), was most consistent in correlating with EVT-3 scores across all tasks. Moreover, for Pearson correlation, this strong association holds for within the ASD-group, but only for the single-word tasks. Overall, the DDK task, a measure that is also used clinically, generally provided the highest associations between the complexity index and the standardized clinical assessments for all three subsystems, followed by Imitation and Naming, which showed varying associations mainly with articulatory and respiratory subsystems, respectively. At the group level, envelope velocity for the DDK task provided some of strongest associations with EVT-3 and PPVT-5 across all clinical measures. Other alternate features beyond base, especially for the laryngeal subsystem, also provided robust association with clinical scores.

## Discussion

This paper lays the foundation and framework for characterizing the movement dynamics of respiratory, laryngeal, and articulatory speech subsystems of a mv-ASD population relative to a neurotypical (NT) group. The complexity of dynamics was determined in the context of each subsystem viewed as a dynamical system, and was reduced to a single variable based on the eigenspectrum of a correlation matrix. Initial results manifest potential connections of acoustic features with clinical assessments of language, cognitive, and fine motor skills. Though results based on the speech data from the 27 mv-ASDs and 27 NTs suggest insightful group-level effect sizes and correlations, the findings also highlight subject-, feature-, subsystem- and task-dependence of motor complexity, not unexpected in light of the large heterogeneity of the ASD population and in particular of mv-ASD individuals further supported in this study. As such this effort provides a ‘tip-of-the-iceberg’ investigation toward phenotyping and personalized intervention, but presents a useful conceptual and quantitative framework for future analysis of data from mv-ASD.

Our results prompt exploring interpretations in light of theoretical frameworks about ASD, speech and motor development. We found *reduced complexity* of articulatory and laryngeal subsystems in DDK for individuals with mv-ASD. This appears to be in keeping with known difficulties with motor skills in mv-ASD and the speech motor demands of DDK, viz. rapid oral repetition of a single syllables (pa, ta, ka), or rapid oral repetition of a sequence of syllables (pa-ta-ka) requiring alternating between syllables with different places of articulation (labial, alveolar and velar), within a single breath. In contrast, we found *increased complexity* in the articulatory system for single words, suggesting great variability and inconsistency in word production in mv-ASD. We speculate that complexity here may be capturing a qualitatively different type of demand on the motor system; one that is mediated by language, given the subjects’ familiarity with the target words. As such, linguistic effects may be influencing the coordination of speech motor gestures. That complexity was *reduced* in the laryngeal and respiratory systems with words also aligns with a reported lack of affect and monotone quality of speech in mv-ASD. It may well be that early speech production difficulties disrupt the development of robust speech representations (Goodell and Studdert-Kennedy, 1993), and have cascading effects on coordination within and between speech subsystems in mv-ASD and other developmental speech sound conditions. This may also explain the correlations between fine motor skills and expressive vocabulary found in a related study (Pecukonis et al., 2025).

Below we outline some on-going and future directions, together with limitations of the study.

### Data quantity and breadth

A future effort should expand not only the number of mv-ASDs in the sample but also include a broader range of spoken language abilities. A larger sample would support stronger group statistics as well as the discovery of clusters (phenotyping) of motor dynamics across the mv-ASD population. One approach to obtaining such a sample size is through at-home collections such as with the ReCANVo database of real-world communicative and affective nonverbal vocalizations (Johnson et al., 2023; Talkar et al., 2024a). On the other hand, we also seek to deepen our understanding of outliers (e.g., those who are non-speaking) and the individualized nature of such cases to motivate personalized intervention. In spite of the relatively small sample, the dataset we have collected is rich in its breadth and depth, and the findings described in this paper open a world of options. For example, investigating the single syllable and word level, and comparisons with core words vs. all combined words, may reveal new insights about the role of vocabulary and top-down effects on speech production across mv-ASD participants. At a finer level is exploring the nature of the single-word tasks and word selections. These considerations may help in separating the effects of motor- vs. linguistic (cognitive)-based tasks.

### Feature selection

In this paper, we chose to focus on 5 features, 3 base and 2 alternate, each feature trajectory associated with movement dynamics of specific subsystems, as well as velocities of base feature trajectories. There are a host of refinements and other feature options to explore in future work. For example, acoustic-to-articulatory inversion (Espy-Wilson et al., 2019; Sivaraman et al., 2017) is an approach to explicitly model the movements of the articulatory subsystem during speech. In this way, correlation-based measurements more accurately represent the complexity of the underlying motor system, as opposed to representing the complexity of proxy signals. As we develop this work further, we would like to maintain the off-body data collection system and use existing or develop new inversion techniques to represent the underlying movements of complete aspects of all relevant motor systems. For example, we could use inversion techniques to estimate movement of the velum in the articulatory subsystem (Siriwardena et al., 2023) and glottal openings and closings and subglottal pressure of the laryngeal and respiratory subsystems, respectively (Chien et al., 2017) to obtain a more accurate or complete feature representation of the subsystems.

As an example, and as noted in our Feature Foundation section, atypical variation in a waveform envelope does not necessarily represent a respiratory system component alone. (Hence, why we chose to use loosely the term ‘respiratory.’) We might imagine a bursty nature of an utterance stems in-part from building up air behind the lips (as in /pa/) or tongue (as in /ta/ and /ka/), and an unnatural release of the lips or tongue during the stop/release pattern that causes the burst as opposed to control that stems from the lungs. Acoustic-to-articulatory inversion may reveal this articulatory coordination issue. Inversion may also help in separating yet other non-respiratory components of the envelope due to harmonic/formant interaction (Horwitz-Martin et al., 2016) or changes in the hardness of glottal closure (Zhang, 2016).

It is important to note that our features were originally developed and validated on verbal utterances of NT adults. Consequently, this raises the possibility of a feature bias such that what we are detecting is the difference in the algorithm’s output as opposed to detecting actual physiological differences between the two groups, a challenge that occurs more generally in analysis of disordered speech in clinical samples (Ramanarayanan et al., 2022). This highlights the importance of keeping in mind clinical consideration when using our approach (Croft et al., 2025).

### Cross-subsystem and multi-modal relations

Our focus in this paper was on movement dynamics within each subsystem. However, we have yet to expand to the coordinated control of dynamics across subsystems such as articulation with phonation, and respiration with phonation (Gracco and Löfqvist, 1994; Gramming et al., 1988). In addition to across speech subsystems, it will also be important to analyze the relationship across modalities to determine the complexity of interactions between speech subsystems and other fine-motor systems, including hand gestures and ocular movements. For example, facial expression may be tightly coupled to articulation and laryngeal movements during speaking. This could give us further insight into how the coupling between subsystems affects performance on a task, and whether any potential deficits seen in the performance on a task stem from one subsystem alone, more than one subsystem or the interaction of subsystems, keeping in mind the influence of cognitive and intellectual abilities on motor performance.

Such efforts should aim to discover the relation of speech motor performance with other fine motor skills (facial expression, eye tracking and hand dexterity) and correlations of our measures with additional clinical assessments such as social skills, and receptive language, as further foundation for developing personalized intervention for communication enhancement.

### Nature of complexity

There are many potential causes for a set of signals to have higher complexity as compared to another – added noise, higher frequency, and a phase shift (Talkar, 2023). While we have identified these causes, we have not yet quantified how each of these modifications are represented in the signals of our sample. The creation of a computational model that determines the degree to which each modification contributes to the signals in mv-ASD could help in better understanding what is happening to the underlying movement dynamics to cause lower or higher complexity. Moreover, we have investigated only one way to define ‘complexity’ as degrees of freedom associated with the volume (e.g., flat or dispersed) of an eigenvalue basis. In addition, toward reducing sensitivity to positive or negative low-energy noisy eigenvalues, we did not include those of high rank and used the real log component. This may not be the most appropriate approach to characterize complexity of movement dynamics in our context where the systems being addressed are potentially nonlinear and it’s difficult to know the importance of low-energy eigenvalues. It behooves us then to compare our measure with other complexity measures such as entropy *Σ* [eigenvalues ∗ log(eigenvalues)], a dimension measure sometimes used to characterize nonlinear dynamical systems *Σ* [eigenvalues^2^], or a dominant number of principle components of the eigenspectrum needed to ‘explain’ the data.

### Translation

An understanding of the challenges of minimally-verbal individuals in each of the three speech production subsystems - respiratory, laryngeal, and articulatory - and their correlation with standardized motor, cognitive, and language assessments, could promote the development of targeted personalized interventions. For example, our results suggest that for the mv-ASD group articulation correlations with EVT-3 are relatively high across single-word tasks, thus supporting the notion of intervention based on articulation to improve EVT. The converse may hold true, however, given the experimental support that children first build their word repertoire as integral sequences of gestures and then differentiate these into their segmental components (Macken, 1979). As such, training of gestural sequences that map onto phonological units may in turn help improve articulation, thereby highlighting the bidirectional relationship between articulation and phonological development. In addition, as alluded to in the Methods section on complexity dynamics, it will be important to intervene in a way that seeks the ‘sweet-spot’ between too little or too large a complexity not only for articulation but for each production subsystem. Proper personalized intervention will drive these disordered subsystems to the ‘sweet spot’ of variability. Our goal is to enhance verbal communication, promote verbal communication when it does not exist, and if not possible, use our insights in motor impairment to promote or enhance a different form of communication in individuals with mv-ASD.

The translation of our work into clinically useful metrics, however, is not without challenge given the heterogeneity of speech production in mv-ASD. We hope to work with clinicians to refine our correlation-based measurements through application in prescribed speech production treatment routines toward development of a ‘change’ index for ease of use by clinicians in determining the efficacy of personalized intervention approaches.

## Data Availability

The datasets presented in this article are not readily available because access requires permisson from MGH Lurie Center for Autism and participants. Requests to access the datasets should be directed to Maria Mody.
